# How involuntary subordination and social support influence the association between self-esteem and depression: a moderated mediation model

**DOI:** 10.1186/s12888-019-2330-1

**Published:** 2019-12-11

**Authors:** Qiuming Shen, Yue Shi, Shuxian Zhang, Lhakpa Tsamlag, Huwen Wang, Ruijie Chang, Zihe Peng, Ying Wang, Meili Shang, Yong Cai

**Affiliations:** 1 School of Public Health, School of Medicine, Shanghai Jiao Tong University, No.227, South Chongqing Road, Shanghai, 200025 People’s Republic of China; 2 Zhongnan Hospital of Wuhan University, Wuhan, 430071 People’s Republic of China; 3 Sanlin Community Health Service Center, No.375, Sanlin Road, Shanghai, 200126 People’s Republic of China

**Keywords:** Migrant, Psychology, Depression, Involuntary subordination, Social support

## Abstract

**Background:**

In China, young migrants are at elevated risk of mental health problems, such as depression. The influence of self-esteem on depression is well acknowledged. We examined correlates of depression and their mediating and moderating role in the association between self-esteem and depression to promote a better understanding of depression prevention among young migrants.

**Methods:**

We conducted a cross-sectional study among young Chinese migrants. A moderated mediation model was used to test the combined effect of involuntary subordination and social support on the association between self-esteem and depression. The Johnson–Neyman method was used to identify the range of scores for which social support acted as a moderator.

**Results:**

A total of 572 participants completed questionnaires. The median depression score was 19 (interquartile range: 14). Self-esteem had a negative effect on involuntary subordination (β = − 2.1440, *p* < 0.001). Involuntary subordination (β = 0.2406, p < 0.001), self-esteem (β = − 0.3870, *p* < 0.01), and social support (β = − 0.1221, p < 0.01) all had significant effects on depression. The effect of involuntary subordination on depression was moderated by social support (β = − 0.0041, *p* < 0.05), and the effect decreased as social support scores increased.

**Conclusions:**

Our results indicated a mediating role of involuntary subordination and a moderating role of social support in the association between self-esteem and depression among young Chinese migrants. Future intervention strategies should focus on these factors to reduce depressive symptoms.

## Background

Migration has become inevitable in an increasingly interconnected world [1]. During recent decades, there has been massive internal migration in developing countries. In China, the number of internal migrants has been rising and reached 286 million in 2017, an increase of 4.81 million (1.7%) over the previous year [2]. Mental health problems are a notable health hazard associated with migration [3–5]. A recent systematic review investigating mental health among rural-to-urban migrants found that depression in migrants is more serious than in the general population in China [6]. Similarly, comparison studies have shown that migrants are more likely to have clinically relevant depressive symptoms [7] and have a higher risk of first onset of depressive disorder [8]. Young migrants may be particularly at risk. Research indicates that new generation migrant workers (aged < 32 years) have poorer mental health than their older counterparts (aged ≥32 years) [9]. A study of Mexican migrants in the United States showed that the increased risk of depression was restricted to individuals aged 18–25 years [8]. All these findings highlight the importance of investigation into mental health problems, particularly depression, among young migrants, a high-risk group.

Previous studies have identified several factors, including psychosocial factors, associated with an elevated risk of depression in young migrants. Numerous studies have documented strong concurrent relations between low self-esteem and depression [10]. Studies on the etiology of depression have noted the importance of low self-esteem [11]. Longitudinal studies have shown that low self-esteem predicts subsequent depression, which suggested that self-esteem has a critical causal role in depression etiology [12]. According to the vulnerability model, a dominant explanation of the association between low self-esteem and depression, low self-esteem contributes to the development and maintenance of depression through both intrapersonal and interpersonal pathways [10]. Many migration related factors may have a close relationship to the interpersonal pathway, which includes reassurance-seeking about personal worth, self-verification through negative feedback, and social avoidance [13, 14]. The relatively low socioeconomic status and unsuccessful acculturation process of migrants in a new environment may influence this pathway and thereby increase the risk of depression [15, 16].

Although it is well acknowledged that self-esteem influences depression, the mechanism underlying this effect is unclear. Another factor related to depression is involuntary subordination; however, research on this association has been scarce. According to social rank theory, involuntary subordination is an adaptive mechanism to avoid fighting when loss is imminent or the cost of winning is too high [17]. Migrants who cannot change their situations in cities may adopt involuntary subordination as an alternative strategy; this makes them more likely to experience a sense of defeat [18] (a sense of failed struggle and losing rank), submissiveness [19] (submissive behaviors), poor social comparisons [20] (feelings of perceived inferiority), and entrapment [17] (feeling stuck or trapped and wanting to escape). However, this strategy should be switched off in time; failure to terminate may result in mood problems such as depression. In an investigation of the psychological aspects of involuntary subordination, Sturman and Mongrain found that involuntary subordination was linked to past episodes of major depression and current depressive symptoms, and predicted recurrence of depression [21, 22]. One study further indicated that involuntary subordination fully mediated the relationship between self-esteem and depressive symptoms [17]. However, no studies have confirmed this conclusion. Therefore, the precise relationship among involuntary subordination, self-esteem, and depression remains to be clarified. As there are multiple causes for most psychological phenomena, a more realistic goal may be to assume that involuntary subordination is a mediator that substantially reduces the association between self-esteem and depression (partial mediation) rather than eliminating the association altogether (full mediation).

Social support is a protective factor for the development of depressive symptoms [23]. Social support has been defined as instrumental, informational, and emotional support provided by a social network; it can protect psychological well-being by buffering the effects of traumatic life events [24]. Research on migrants has identified several stressful events that may threaten mental health, such as separation from family, discrimination, and loss of social status [25]. Under such conditions, inadequate social support may increase the negative impact of a stressful environment. A wide range of studies have shown that social support is negatively related to depression, an association mostly explained by the buffering effect of support on the negative impact of stress on depression [26, 27]. There is evidence that social support moderates the association between traumatic life events and depression in migrants [28]. However, it remains to be established whether this moderation effect applies to psychological problems (e.g., low self-esteem and involuntary subordination) caused by traumatic life events. It is therefore necessary to examine whether social support moderates the associations among self-esteem, involuntary subordination, and depression.

**Fig. 1:**
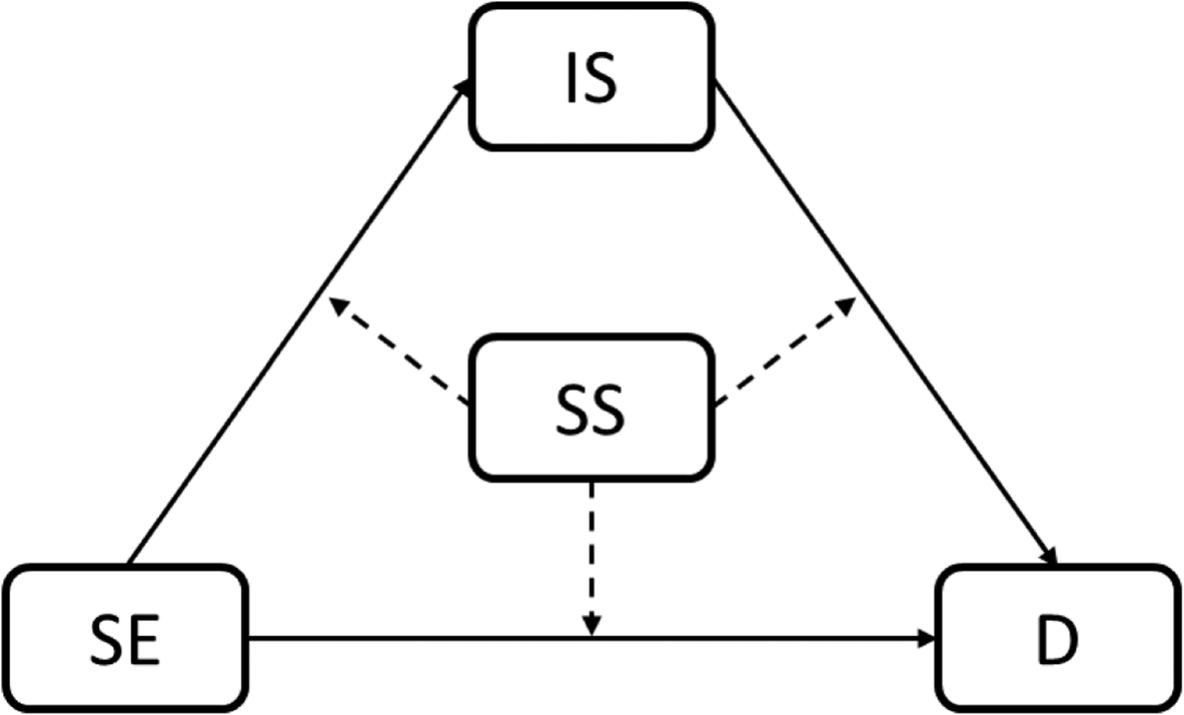
The proposed moderated mediation model

As mentioned above, involuntary subordination and social support may play important roles in the association between self-esteem and depression, but the possible influence of these mechanisms is unclear. Moderated mediation combines mediation and moderation to determine if the strength of an indirect effect is estimated to depend on the level of some variable [29, 30]. The study aim was to examine the mediating role of involuntary subordination and the moderating role of social support in the association between self-esteem and depression among young migrants in China. Our hypotheses were as follows:

1. Depression would be significantly positively associated with involuntary subordination and negatively associated with self-esteem and social support.
2. Involuntary subordination would mediate the relationship between self-esteem and depression (Fig. 1).
3. Social support would moderate the direct and/or indirect effect of self-esteem on depression (Fig. 1).
4. The effect of self-esteem on depression through involuntary subordination would be a decreasing function of social support.

## Methods

### Participants and procedures

The ethics committee of the School of Public Health of Shanghai Jiao Tong University approved the study (SJUPN-201302). This cross-sectional study was conducted from June 2017 to December 2017 with young migrant workers at industrial factories in Shanghai, one of the biggest cities in China. One factory located in the city center and another located in a suburban area were selected with the assistance of local centers for disease control and prevention. With the help of factory managers, convenience sampling was conducted to select individuals who met the inclusion criteria from each factory. Individuals were eligible if they were aged 18–25 years and did not have local household registrations.

We reviewed previous literature and found that reported standard deviations of scores on the Center for Epidemiologic Studies Depression Scale (CES-D) for young Chinese migrants varied from 7.2 to 9.8 [6]. Assuming an estimated standard deviation of 9.5, a distance from the mean to the limits of 0.8, and α of 0.05, we calculated a required sample size of 545. We recruited participants until the required sample size was reached. A total of 830 young Chinese migrants were recruited; 214 of those declined to participate. Finally 572 participants completed the study questionnaire adequately for further analysis.

Data were collected using an anonymous, self-report questionnaire. We obtained written informed consent from all participants following clarification of the study objectives and procedure, as well as the potential risks and benefits of participation. Participants completed the questionnaire in a private room. One investigator was present to answer any questions. The questionnaire was completed in approximately 30 min and respondents were given 50 Chinese yuan (approximately 7 United States dollar) for participation after satisfactorily completing the survey.

### Measures

#### Sociodemographic variables

The following sociodemographic information was collected: age, gender, education, marital status, income level, and length of time working in Shanghai.

#### Depression

Depressive symptoms were measured using the 20-item Center for Epidemiologic Studies Depression Scale (CES-D) [31]. Responses are rated on a Likert-type scale comprising 16 positive-scoring and 4 reverse-scoring items. Participants are asked how often they have experienced depressive symptoms within the past week; 0 = rarely or none of the time/less than 1 day, and 3 = most or all of the time/5–7 days. The total possible score ranges from 0 to 60; higher summed scores indicate greater depression severity. The Chinese version of CES-D has shown good validity and reliability for the general Chinese population [32]. In this study, Cronbach’s α was 0.871. The average inter–item correlation was 0.331 and the item–total correlations ranged from 0.386 to 0.713.

#### Self-esteem

Self-esteem was measured using the 10-item Rosenberg Self-Esteem Scale (RSES) [33, 34]. Responses are rated on a Likert-type scale. Five items are positively worded and five are negatively worded. Participants are asked to rate how much they agree or disagree with each item; 0 = strongly disagree and 3 = strongly agree. Positively worded responses are reverse-scored so that all item response scores can be summed. The total possible score ranges from 0 to 30; higher total scores indicate lower self-esteem. The Chinese version of RSES has shown good levels of reliability and validity for the Chinese population [35, 36]. In this study, Cronbach’s α was 0.649. The average inter–item correlation was 0.309 and item–total correlations ranged from 0.389 to 0.687.

#### Involuntary subordination

Involuntary subordination was measured using the 32-item Involuntary Subordination Questionnaire [17]. Responses are rated on a 5-point Likert scale; 1 = strongly disagree and 5 = strongly agree. The total possible score ranges from 32 to 160; higher total scores indicates higher levels of involuntary subordination. The Chinese version has been used with men who have sex with men and has shown sufficient reliability [37]. In this study, Cronbach’s α was 0.895. The average inter–item correlation was 0.309 and the item–total correlation ranged from 0.300 to 0.717.

#### Social support

Social support was measured using the 12-item Multidimensional Scale of Perceived Social Support (MSPSS) [38]. Items are scored on a 7-point Likert scale; 1 = very strongly disagree and 7 = very strongly agree. The total possible score ranges from 7 to 84; higher scores indicate greater social support. The Chinese version of the MSPSS has shown good validity and reliability for the Chinese population [39, 40]. In this study, Cronbach’s α was 0.920. The average inter–item correlation was 0.501 and the item-total correlation ranged from 0.665 to 0.780.

### Statistical analyses

SPSS 22.0 (IBM Corp. Released 2013. IBM SPSS Statistics for Windows, Version 22.0. Armonk, NY, USA) was used for data cleaning, coding, and preliminary analysis. In cases where data from a participant were missing for a particular variable, the conservative approach of listwise deletion was used, and the participant’s data were completely excluded from the analysis. Descriptive statistics including medians and interquartile ranges (Q) were used for continuous variables, as the main study variables were not normally distributed. Numbers and percentages were used for binary and categorical variables. Spearman correlation was used to evaluate the correlations between variables. The mediation model and moderated mediation model were analyzed using the PROCESS macro for SPSS [29]. All continuous variables were standardized and the interaction terms were computed from these standardized scores. The bias-corrected 95% confidence interval (CI) was calculated with 5000 bootstrapping samples. To identify the statistically significant region, or range of scores for which social support acted as a moderator, the Johnson–Neyman method suggested by Hayes [29] was used. The conditional effects and CI were plotted.

## Results

### Description of participants

In total, 616 participants completed the questionnaires; 572 questionnaires remained after deletion owing to missing variables. Table 1 shows the participant characteristics. As discussed previously, all participants were young adults, with a mean age of 21.61 years (SD = 0.07, range 18.30–23.40). Most were male (61.89%); 64.34% had a highest education level of senior high school; 82.17% were unmarried; most of the sample had a monthly income of 3200–4800 Chinese yuan (64.51%); and 61.01% had been working in Shanghai for less than 1 year. There were no significant differences in depression scores by participant characteristics.

**Table 1:** Participant sociodemographic characteristics (*N* = 572)

Characteristic variables	Number (%)	Depression symptoms Median(Q)	Z/H	p
*Gender*			−1.238	0.216
male	354 (61.89)	18 (15)		
female	218 (38.11)	19 (14)		
*Education*			0.643	0.725
Junior high school and below	137 (23.95)	18 (15)		
Senior high school	368 (64.34)	19 (14)		
College and above	67 (11.71)	17 (13)		
*Current marital status*			−0.834	0.404
Unmarried	470 (82.17)	19 (14)		
Married	102 (17.83)	18 (14)		
*Average monthly income level (Chinese Yuan)*			1.387	0.500
< 3200	168 (29.37)	19 (18)		
3200–4800	369 (64.51)	18 (14)		
> 4800	35 (6.12)	18 (11)		
*Length of time working in Shanghai*			1.684	0.431
< 1 year	349 (61.01)	19 (16)		
1–5 years	204 (35.66)	18 (13)		
> 5 years	19 (3.32)	18 (17)		

*Q*: interquartile range

### Description and correlation of main study variables

The mean (SD) scores for self-esteem, involuntary subordination, social support, and depression were 18.30 (3.99), 83.48 (15.66), 63.90 (12.38), and 19.18 (10.06), respectively. The median (Q) scores for self-esteem, involuntary subordination, social support, and depression were 18 (4), 82 (18), 66 (16), and 19 (14), respectively (Table 2).

**Table 2:** Descriptive statistics and correlations of the main study variables

	Median	Q	I	II	III	IV
I Self-esteem	18	4	1			
(Range: 6–30)						
II Involuntary subordination	82	18	−0.608^*^	1		
(Range: 32–128)						
III Social support	66	16	0.427^*^	−0.508^*^	1	
(Range: 12–84)						
IV Depression	19	14	−0.431^*^	0.520^*^	−0.401^*^	1
(Range: 0–49)						

Q: interquartile range

*p < 0.001

Migrants with higher levels of self-esteem and higher levels of social support were more likely to have lower levels of involuntary subordination and depression. Those who had higher levels of involuntary subordination were more likely to have depressive symptoms.

Our results also showed that involuntary subordination correlated strongly (r > 0.5, *p* < 0.001) with both self-esteem and depression, whereas social support was moderately (r = 0.3–0.5, p < 0.001) correlated with self-esteem and depression.

### Testing for mediation (involuntary subordination as a mediator)

Table 3 shows the mediating role of involuntary subordination in the association between self-esteem and depression. In Model 1 and Model 2, self-esteem had a negative effect on both depression (β = − 1.1465, *p* < 0.001) and involuntary subordination (β = − 2.5153, *p* < 0.001). In Model 3, self-esteem had a negative effect (β = − 0.4394, *p* < 0.01) on depression, and involuntary subordination (β = 0.2811, p < 0.001) had a positive effect on depression.

**Table 3:** Mediating role of involuntary subordination in association between self-esteem and depression

predictors	Model1(Y = D)		Model2(Y=IS)		Model3(Y = D)	
	β	BC 95% bootstrapped CI	β	BC 95% bootstrapped CI	β	BC 95% bootstrapped CI
SE	−1.1465***	(−1.3310,-0.9616)	−2.5153***	(−2.7635, − 2.2670)	− 0.4394**	(− 0.6627, − 0.2160)
IS					0.2811***	(0.2242, 0.3380)
F	148.264***		396.0725***		133.3636***	
R^2^	0.2070		0.4108		0.3199	

**p < 0.01, ***p < 0.001

*SE* Self-esteem, *IS* Involuntary subordination, *D* Depression, *CI* Confidence interval

BC: Confidence intervals were bias-corrected

The size of the mediation effect of involuntary subordination was − 0.7071. The direct effect from self-esteem to depression was − 0.4394. The indirect effect/direct effect was 1.6092.

### Testing for moderated mediation (involuntary subordination as a mediator; social support as a moderator)

Model 4 showed that self-esteem (β = − 0.9041, *p* < 0.001) and social support (β = − 0.1871, p < 0.001) were significant predictors of depression. Model 4 showed no moderation of the overall effect (β = 0.0122, *p* > 0.05).

Model 5 yielded a significant effect of self-esteem (β = − 2.1440, p < 0.001) and social support (β = − 0.2867, *p* < 0.001) on involuntary subordination. There was no moderation effect for the interaction between self-esteem and social support (β = − 0.0116, p > 0.05).

As the overall effect of self-esteem on depression was not moderated by social support, Model 6 did not include the moderation effects of the interaction between self-esteem and social support. The analysis indicated a significant effect of self-esteem (β = − 0.3870, *p* < 0.01), social support (β = − 0.1221, p < 0.01), and involuntary subordination (β = 0.2406, p < 0.001) on depression. The effect of involuntary subordination on depression (the latter path of the indirect effect) was moderated by social support (β = − 0.0041, *p* < 0.05).

**Fig. 2:**
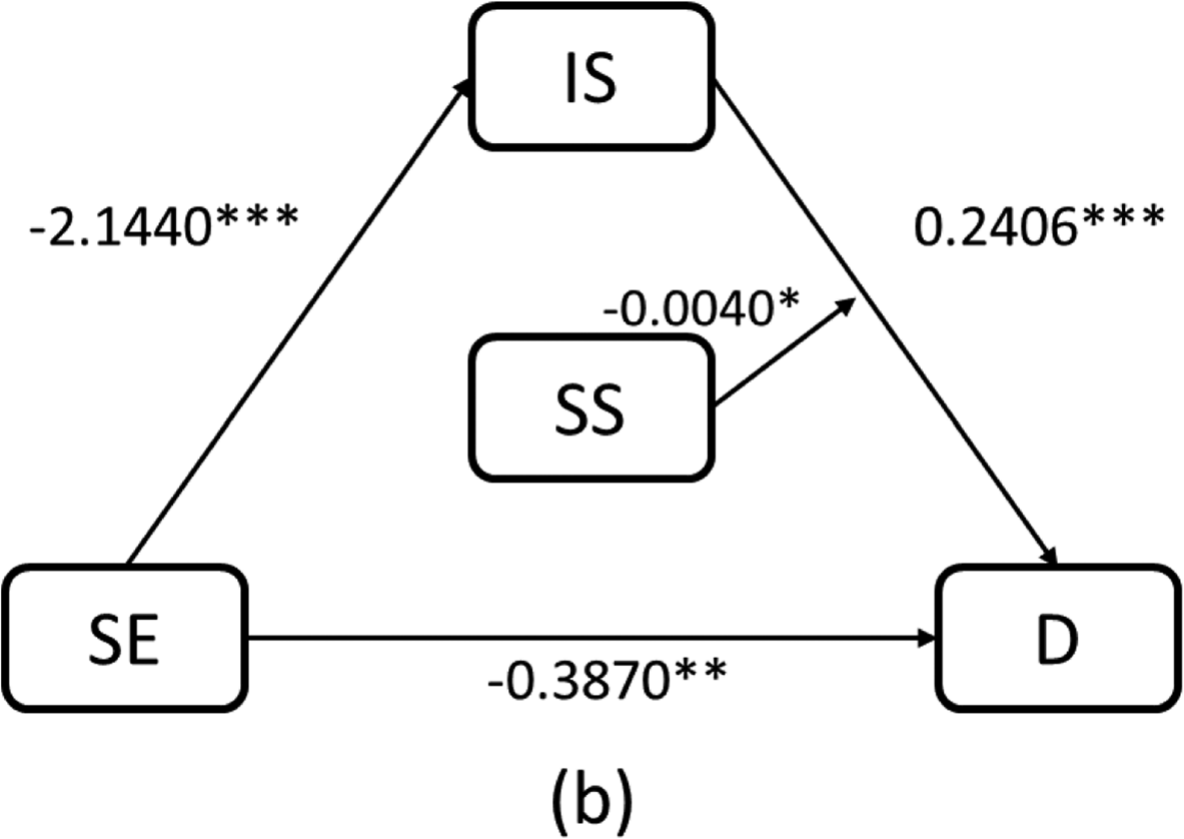
Schematic model of social support as a moderator of the mediation model

Overall, involuntary subordination mediated the effect of self-esteem on depression and social support moderated the effect of involuntary subordination on depression. The R square of the moderated mediation model was 0.3426. The final pattern is shown in Fig. 2 and its test results are summarized in Table 4.

**Table 4:** Mediating role of involuntary subordination and moderating role of social support in association between self-esteem and depression

predictors	Model4(Y = D)		Model5(Y=IS)		Model6(Y = D)	
	β	BC 95% bootstrapped CI	β	BC 95% bootstrapped CI	β	BC 95% bootstrapped CI
SE	−0.9041***	(− 1.1015, − 0.7067)	−2.1440***	(−2.4080, − 1.8801)	−0.3870**	(− 0.6128, − 0.1613)
SS	−0.1871***	(− 0.2511, − 0.1232)	−0.2867***	(− 0.3722,- 0.2013)	−0.1221**	(− 0.1843, − 0.0600)
IS					0.2406***	(0.1817, 0.2995)
SE*SS	0.0122	(−0.0012, 0.0265)	−0.0116	(− 0.0295, 0.0063)		
IS*SS					−0.0040*	(−0.0078, −.0003)
F	65.5453***		156.3184***		73.6014***	
R^2^	0.2578		0.4531		0.3426	

*p < 0.05, **p < 0.01, ***p < 0.001

*SE* Self-esteem, *IS* Involuntary subordination, *D* Depression, *SS* Social support, *CI* Confidence interval

BC: Confidence intervals were bias-corrected

### Testing for a conditional effect

**Fig. 3:**
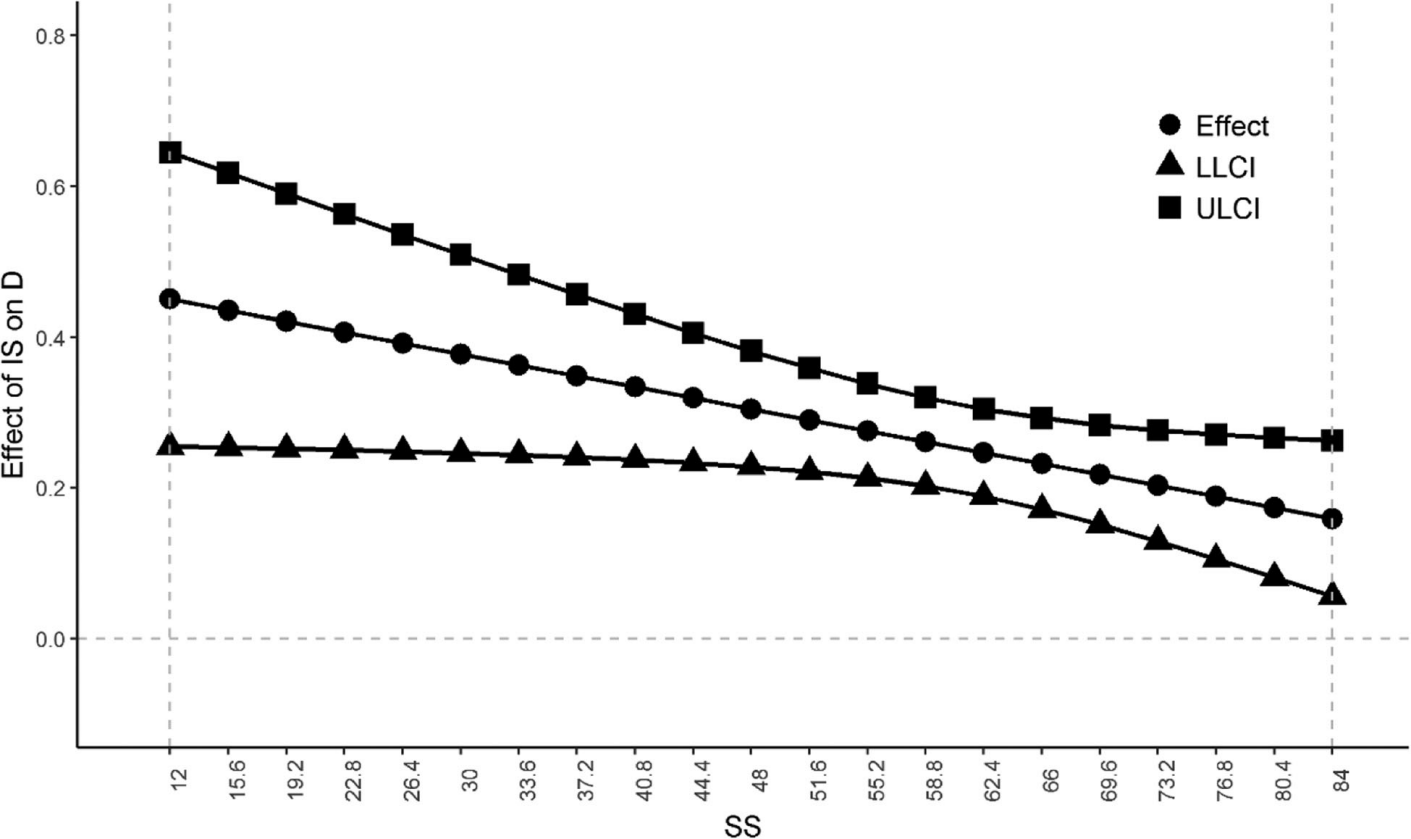
Conditional effect of involuntary subordination on depression by social support values

Following determination of a significant interaction, the estimated conditional effects of involuntary subordination on depression at different levels of social support were examined. The interaction effect was significantly moderated throughout the observed range of social support (12–84). As social support scores increased, the conditional effect of involuntary subordination on depression decreased (range: 0.1594–0.4504, effect of involuntary subordination on depression at mean level of social support: 0.2406). Figure 3 shows the conditional effect at different social support scores.

To further validate the moderated mediation relationship, we examined conditional indirect effects. As shown in Table 5, the conditional indirect effect of self-esteem on depression through involuntary subordination was weaker among participants who scored higher on social support (social support = 51.5217, indirect effect = − 0.5815; social support = 63.9000, indirect effect = − 0.5159; social support = 76.2783, indirect effect = − 0.4359).

**Table 5:** Conditional indirect effects of self-esteem on depression

SS	Indirect Effect	BC 95% bootstrapped CI
Mean-1SD(−12.3926)	−0.5815	(− 0.7777,-0.4081)
Mean(− 0.0143)	−0.5159	(− 0.6643,-0.3796)
Mean + 1SD(12.3640)	−0.4359	(− 0.6326,-0. 2520)

Confidence intervals that did not contain zero values were considered significant

*M* Mean, *SD* Standard deviation, *SS* Social support, *CI* Confidence interval

BC: Confidence intervals were bias-corrected

## Discussion

The combined effect of mediation and moderation has seldom been considered when studying the relationship between self-esteem and depression among young Chinese migrants. This study established a moderated mediation model to test these underlying mechanisms. Our results showed that involuntary subordination mediated the relationship between self-esteem and depression, and social support moderated the latter path of the indirect effect. The conditional indirect effect was weaker for participants who scored higher on social support.

### Depression among young Chinese migrants

Our findings confirmed the vulnerability of young Chinese migrants to depression. Their Center for Epidemiologic Studies Depression Scale score (19.18 [10.06]) was higher than the norm for the general Chinese population (13.24 [10.33]) and the norm of a relatively young Chinese population aged 18–30 years (12.32 [10.22]) (mean[SD]) [32]. These findings are consistent with the conclusion of a meta-analysis that the depression status of migrants is more serious than that of the general population in China [6].

### The mediating role of involuntary subordination

As expected, the findings showed that involuntary subordination mediates the relationship between self-esteem and depression. Involuntary subordination was negatively associated with self-esteem, a finding in accord with several previous studies [17, 41]. Research indicates that self-appraisal, which includes beliefs about self-worth, may have a direct impact upon situational appraisal [42]. For young migrants, stressful events during migration are strongly associated with the development of negative appraisals [43]. Migrants with high self-esteem are likely to form positive self-appraisals, which reduces the likelihood that stressful events will be negatively appraised (situational appraisal). Conversely, when self-appraisals are negative, stressful events may be negatively appraised and cause feelings of defeat and entrapment [42]. Social rank theory suggests that a sense of being of a lower rank than others is likely to cause depression [44]. The present findings confirm this assumption and demonstrate a positive association between involuntary subordination and depression. Most young migrants are unable to alter unfavorable situations in big cities and have to face challenges such as unsuccessful acculturation, discrimination, and low socioeconomic status. Therefore, many are likely to experience involuntary subordination regarding their current situation [45]. Previous research indicates that this type of submissiveness is moderately associated with depression in normal populations and strongly associated with depression in depressed populations [46]. Depression may represent the activation of an internal inhibitory system under conditions of perceived involuntary, subordinate status and self-evaluation [47, 48]. The present findings may help to highlight the long-neglected role of involuntary subordination in the relationship between self-esteem and depression.

### The role of self-esteem as an independent predictor

In the mediation model, both self-esteem and involuntary subordination emerged as significant predictors of depressive symptoms. Early studies reported that involuntary subordination is a full mediator [17]. However, this study indicated that involuntary subordination partially mediates the relationship between self-esteem and depression, indicating an independent role of self-esteem. The current findings support the notion that self-esteem is negatively associated with depression. Price has explained this relationship by suggesting that low self-esteem may both trigger an episode of depression and increase vulnerability to depression [49]. Price has argued that mood changes can be regarded as a self-esteem management mechanism in which self-esteem is regulated to fit with current social circumstances [50]. The present findings suggest that the mediation model can explain the effect of self-esteem on depression, and that more focus is needed on the role of both self-esteem and involuntary subordination.

### The moderating role of social support

Social support significantly moderated the effect of involuntary subordination on depression (the latter path) throughout the observed range of social support. As social support increased, the effect of involuntary subordination on depression monotonically decreased. According to the buffering model, the stress response can be modulated by the social environment. Adequate rescue resources can largely mitigate negative social rank and stress response and alleviate feelings of entrapment [44]. Perceived adequate support from family, friends, and significant others provides young migrants with emotional comfort, helps them to cope with stress more easily, and facilitates more successful adjustment to the new environment [51, 52], which in turn mitigates the effect of involuntary subordination on depression. Contrary to expectations, social support did not moderate the effect of self-esteem on involuntary subordination (the former path). This may be explained by the nature of the dependent variable, involuntary subordination, in young migrants. Migrants are often considered to be of a lower rank than local citizens. This is an aspect of the societal context that may not be easily affected by an interpersonal factor like social support. Overall, the present results complement previous findings that social support moderates the association between traumatic life events and depression [28] and demonstrate that social support can further moderate the relationship between psychological problems caused by traumatic life events and depression. Although it is not easy for young migrants to withstand the harsh conditions of migration, sufficient social support can reduce the adverse psychological consequences of involuntary subordination, such as depression [53].

### The role of social support as an independent predictor

The present study also showed that social support affects depression directly. Young migrants with greater social support were less likely to experience depression. This is fairly consistent with previous findings that social support, rather than merely protecting an individual against the negative impact of stress, may itself be important in ameliorating depressive symptoms [53]. More attention needs to be paid to this added benefit of improving social support, as this strategy may simultaneously prevent low self-esteem in young migrants who experience involuntary subordination and subsequent depression, and also directly protect against depression.

### Direct and indirect effects

Consistent with previous research findings [17], the present results revealed that the indirect effect of social support was larger than the direct effect, indicating a dominant role for the indirect effect. Variables that affected the strength of the indirect effect were more important: the impact of self-esteem on depression depended upon the level of social support, indicating that low social support amplified the indirect effect. According to the moderated mediation model, young migrants with lower perceived social support were more vulnerable to the negative effect of low self-esteem on depression through involuntary subordination. This finding provides a theoretical basis for developing interventions to improve social support to prevent depression among young migrants. Social support can be regarded as a defensive mechanism in the indirect effect of self-esteem on depression, and is particularly important, as involuntary subordination is difficult to prevent and to change.

### Synthesis of results from the moderated mediation model

The model tested here combined mediation and moderation to provide a new understanding of factors that correlate with depression. Previous researches indicated that low self-esteem played a critical causal role in the etiology of depression. On this basis, we introduced involuntary subordination, a long-neglected factor, to the association between self-esteem and depression. This is the first study to confirm its mediating role in the relationship between self-esteem and depression. We also considered the moderating role of social support on depression and determined how this factor influenced the indirect effect of self-esteem on depression. These findings may inform strategies for depression prevention both theoretically (in understanding how these variables affect depression) and practically (the development of interventions such as social support).

Although the R square of the moderated mediation model was not high, we believe it was sufficient for a model with only three main variables. R square has a close relation to the number of independent variables (the more independent variables that the model contains, the higher R square will be). We focused on the role of involuntary subordination and social support in the association between self-esteem and depression. Therefore, we did not include other factors, like stress, in our model. Furthermore, there were no differences in depression status between gender or income groups. As a result, sociodemographic characteristics were not included in the model as controls. Despite the limited number of independent variables in the moderated mediation model, the proportion of variance explained by the model was sufficient.

The dataset used for analysis excluded cases with missing data (*N* = 572). We tested the moderated mediation model again using imputation for missing data (*N* = 616). The results showed similar findings (involuntary subordination mediated the association between self-esteem and depression; social support moderated the latter path of the indirect effect), which indicated the robustness of model (see Additional file 1).

Considering the proportion of variance explained by the model and the robustness of the non-imputation and imputation datasets, we suggest that this moderated mediation model will be useful for future studies of depression.

### Strengths and limitations

The study strengths include the use of a sample of young migrants, the focus on the long-neglected role of involuntary subordination, further exploration of the role of social support, and consideration of the combined effects of mediation and moderation.

Our findings should also be interpreted with caution owing to methodological limitations of the study. The cross-sectional design makes it difficult to establish cause–effect relationships. The initial hypothesized cause–effect relationships were based on previous research findings. Additionally, the sample was drawn from two factories and so may not be representative of all young migrants in the region. It was difficult to obtain a random sample as migrants are generally mobile. Therefore, we used convenience sampling, which limited the representativeness of the sample. Another limitation is that self-report data are subject to possible bias. This is particularly the case for measures of psychological variables, which need to be culturally sensitive owing to differences in how psychological well-being is constructed and reported. We minimized this problem by using scales with good validity and reliability for the Chinese population, using anonymous questionnaires, and providing private rooms for the survey. Finally, there was no control group of non-migrants. Therefore, there is a possibility of bias in this study.

## Conclusion

This study elucidated the ways in which self-esteem leads to depression. The results indicate that young Chinese migrants are at greater risk of depression than the general population. Involuntary subordination played a mediating role in the relationship between self-esteem and depression. Additionally, the strength of the indirect effect was moderated by social support: greater social support reduced the effect of involuntary subordination on depression. To reduce depression in young migrants with low self-esteem, particularly those with high involuntary subordination and low social support, interventions that both improve involuntary subordination and increase social support are needed.

## Supplementary information

**Additional file 1:** Results of analysis of imputation dataset.

## Data Availability

The datasets generated and analyzed during the current study are not publicly available due to the funding nature (National Natural Science Foundation of China) but are available from the corresponding author on reasonable request.

## Abbreviations

BC: Bias-corrected
CES-D: Center for Epidemiologic Studies Depression Scale
CI: Confidence interval
D: Depression
IS: Involuntary subordination
LLCI: Lower-limit confidence interval
MSPSS: Multidimensional Scale of Perceived Social Support
Q: interquartile ranges
RSES: Rosenberg Self-Esteem Scale
SD: Standard deviation
SE: Self-esteem
SS: Social support
ULCI: Upper-limit confidence interval
