# Supplementary material for: How involuntary subordination and social support influence the association between self-esteem and depression: a moderated mediation model
Source: BMC Psychiatry. 2019 Dec 11;19:390. doi: 10.1186/s12888-019-2330-1 (PMC6907199; doi:10.1186/s12888-019-2330-1)
Supplement: Supplementary file 1 — Additional file 1: Results of analysis of imputation dataset. [file 12888_2019_2330_MOESM1_ESM.docx]

Appendix: results of analysis of imputation dataset

**Description about missing data**

There were altogether 44 participants with missing data (7.14% of all participants who completed the questionnaires).

All of the missing were item missing. We requested investigators to look through the questionnaires after participants finished answering (not for the content but for better completion rate). Therefore, there is no condition like missing a whole scale or a large part of a scale. Most missing conditions were one or two item missing in the middle of a scale that cannot be noticed by investigators’ quick glances.

**Imputation method**

For item missing, we imputed the data by referring to the nearest related item. For example, there was a one missing data in the involuntary subordination scale. Involuntary subordination scale consist of items measuring defeat, submissive behavior, ect. The missing data (item 1) measured defeat. Therefore, we imputed data with reference to the nearest item that also measured defeat (item 5).

**Results of analysis of imputation dataset**

The results showed similar findings to the non-imputation dataset.

**Table 1 Participant sociodemographic characteristics (*N* = 616)**

| Characteristic variables | Number | Depression symptoms | Z/H | p |
| --- | --- | --- | --- | --- |
|  | (%) | Median(Q) |  |  |
| *Gender* |  |  | -0.857 | 0.392 |
| male | 388(62.99) | 18(15) |  |  |
| female | 228(37.01) | 19(14) |  |  |
| *Education* |  |  | 0.845 | 0.656 |
| Junior high school and below | 149(24.19) | 18(15) |  |  |
| Senior high school | 396(64.29) | 19(15) |  |  |
| College and above | 71(11.53) | 17(13) |  |  |
| *Current marital status* | |  | -0.940 | 0.347 |
| Unmarried | 514(83.44) | 19(15) |  |  |
| Married | 102(16.56) | 18(14) |  |  |
| *Average monthly income level (Chinese Yuan)* |  |  | 0.169 | 0.919 |
| <3200 | 185(30.03) | 19(18) |  |  |
| 3200-4800 | 390(63.31) | 18(14) |  |  |
| >4800 | 41(6.66) | 19(11) |  |  |
| *Length of time working in Shanghai* |  |  | 1.891 | 0.389 |
| <1 year | 379(61.53) | 19(16) |  |  |
| 1-5 years | 218(35.39) | 18(13) |  |  |
| >5 years | 19(3.08) | 18(17) |  |  |

*Q: interquartile range.

**Table 2 Descriptive statistics and correlations of the main study variables**

|  | Median | Q | I | II | III | IV |
| --- | --- | --- | --- | --- | --- | --- |
| I Self-esteem | 18 | 4 | 1 |  |  |  |
| (Range: 6-30) |  |  |  |  |  |  |
| II Involuntary subordination | 82 | 19 | -0.625^*^ | 1 |  |  |
| (Range: 32-128) |  |  |  |  |  |  |
| III Social support | 66 | 16 | 0.456^*^ | -0.515^*^ | 1 |  |
| (Range: 12-84) |  |  |  |  |  |  |
| IV Depression | 19 | 14 | -0.478^*^ | 0.548^*^ | -0.430^*^ | 1 |
| (Range: 0-49) |  |  |  |  |  |  |

Q: interquartile range.

*p < 0.001.

**Table 3 Mediating role of involuntary subordination in association between self-esteem and depression**

| predictors | Model1(Y=D) | | Model2(Y=IS) | | Model3(Y=D) | |
| --- | --- | --- | --- | --- | --- | --- |
|  | β | BC 95% bootstrapped CI | β | BC 95% bootstrapped CI | β | BC 95% bootstrapped CI |
| SE | -1.2786*** | (-1.4521,-1.1051) | -2.4904*** | (-2.7210, -2.2599) | -0.5587** | (-0.7697, -0.3476) |
| IS |  |  |  |  | 0.2891*** | (0.2340, 0.3442) |
| F | 209.5036*** |  | 450.0684*** |  | 175.7576*** |  |
| R^2^ | 0.2544 |  | 0.4230 |  | 0.3644 |  |

*p < 0.05, **p < 0.01, ***p < 0.001.

SE: Self-esteem; IS: Involuntary subordination; D: Depression; CI: Confidence interval.

BC: Confidence intervals were bias-corrected.

**Table 4 Mediating role of involuntary subordination and moderating role of social support in association between self-esteem and depression**

| predictors | Model4(Y=D) | | Model5(Y=IS) | | Model6(Y=D) | |
| --- | --- | --- | --- | --- | --- | --- |
|  | β | BC 95% bootstrapped CI | β | BC 95% bootstrapped CI | β | BC 95% bootstrapped CI |
| SE | -1.0124*** | (-1.1992, -0.8256) | -2.1077*** | (-2.3561, -1.8593) | -0.5004** | (-0.7141, -0.2868) |
| SS | -0.1922*** | (-0.2542, -0.1303) | -0.2872*** | (-0.3695,- 0.2048) | -0.1296** | (-0.1895, -0.0698) |
| IS |  |  |  |  | 0.2423*** | (0.1852, 0.2994) |
| SE*SS | 0.0158* | (0.0032, 0.0284) | -0.0118 | (-0.0286, 0.0050) |  |  |
| IS*SS |  |  |  |  | -0.0047** | (-0.0081, -.0012) |
| F | 90.9887*** | | 176.7770*** | | 97.5943*** | |
| R^2^ | 0.3084 | | 0.4643 | | 0.3898 | |

*p < 0.05, **p < 0.01, ***p < 0.001.

SE: Self-esteem; IS: Involuntary subordination; D: Depression; SS: Social support; CI: Confidence interval.

BC: Confidence intervals were bias-corrected.

**Table 5 Conditional indirect effects of self-esteem on depression**

| SS | Indirect Effect | BC 95% bootstrapped CI |
| --- | --- | --- |
| Mean-1SD(-12.5668) | -0.5900 | (-0.7838,-0.4210) |
| Mean(-0.0176) | -0.5108 | (-0.6588,-0.3762) |
| Mean+1SD(12.5316) | -0.4141 | (-0.6049,-0. 2414) |

Confidence intervals that did not contain zero values were considered significant.

M: Mean; SD: Standard deviation; SS Social support; CI: Confidence interval.

BC: Confidence intervals were bias-corrected.

**Implications:**

The moderated mediation model showed robustness in our study population.
